# How our hearts beat together: a study on physiological synchronization based on a self-paced joint motor task

**DOI:** 10.1038/s41598-023-39083-9

**Published:** 2023-07-25

**Authors:** Stephan Flory, Sabino Guglielmini, Felix Scholkmann, Valentine L. Marcar, Martin Wolf

**Affiliations:** 1 Biomedical Optics Research Laboratory, Department of Neonatology, University Hospital Zurich, University of Zurich, Zurich, Switzerland; 2 Neurophotonics and Biosignal Processing Research Group, Department of Neonatology, University Hospital Zurich, University of Zurich, Zurich, Switzerland; 3 Institute of Complementary and Integrative Medicine, University of Bern, Bern, Switzerland; 4 Comprehensive Cancer Center Zürich, University Hospital Zürich, Zurich, Switzerland

**Keywords:** Social neuroscience, Cooperation, Empathy, Neuroscience, Sensorimotor processing

## Abstract

Cardiac physiological synchrony is regarded as an important component of social interaction due to its putative role in prosocial behaviour. Yet, the processes underlying physiological synchrony remain unclear. We aim to investigate these processes. 20 dyads (19 men, 21 women, age range 18–35) engaged in a self-paced interpersonal tapping synchronization task under different levels of tapping synchrony due to blocking of sensory communication channels. Applying wavelet transform coherence analysis, significant increases in heart rate synchronization from baseline to task execution were found with no statistically significant difference across conditions. Furthermore, the control analysis, which assessed synchrony between randomly combined dyads of participants showed no difference from the original dyads’ synchrony. We showed that interindividual cardiac physiological synchrony during self-paced synchronized finger tapping resulted from a task-related stimulus equally shared by all individuals. We hypothesize that by applying mental effort to the task, individuals changed into a similar mental state, altering their cardiac regulation. This so-called psychophysiological mode provoked more uniform, less variable fluctuation patterns across all individuals leading to similar heart rate coherence independent of subsequent pairings. With this study, we provide new insights into cardiac physiological synchrony and highlight the importance of appropriate study design and control analysis.

## Introduction

The heart is more than just the organ that maintains and adjusts blood flow to organs: The way we experience life and feel about it constantly influences our hearts and vice versa. The hypotheses that what we experience causes a specific adjustment of our vegetative functions to intensify the simultaneous emotion^1,2^ or that our bodily response to a stimulus even enables us to experience emotion in the first place^3^ have been researched for over a century. One rather special manifestation of this co-dependence of heart and mind manifests itself in social interaction. As social beings humans, we are in constant active or passive interaction with each other. In recent years, the interest about changes in vegetative functions in the context of cognitive and emotional processes extended from a solely intra-individual to an inter-individual perspective. Having a conversation, performing a cooperative task, or simply looking at each other seems to create a special connection between the individuals involved. This manifests in an increased synchronization of several vegetative functions, in particular heart rate (HR)^4–7^. This phenomenon is termed *physiological synchrony* (PS; for a review see Ref.^8^). Higher PS has been associated with multiple prosocial effects, from association to cooperative action^9^, better overall team performance in group tasks^10^, increased group cohesion^11^ to more empathic conversations between a therapist and their client^12^. Even the degree of emotional connection appears to affect PS positively^13^. Generally, PS can be viewed as accompanying shared experiences, yet its true origin and use still remains unknown^8^. Through studying its processes, we, thus, might gain a better understanding of what role PS plays during social interaction.

In search of a task to analyze physiological synchrony in human social interaction, we considered synchronization of motor function as a modality to study. The ability to perceive periodicity and movement in time is elemental for human interactions. This ability has proven itself integral in the way we speak, such as coordinating our vocal cords, which rely on accurately applying the language's rhythmicality^14^. E.g. when working together, it is central to an efficient performance that we time our movements accurately to synchronize them with the movement of others^15^. Every couple who has tried to lift a heavy bookshelf understands the importance of being able to act on the count of three. Thus, it comes as no surprise that there seems to be a certain reciprocity, whereby moving in synchrony tends to positively affect individuals’ affiliation to their surroundings^16^ and to increase cooperation and trust^17^ and in return positive emotions induced by social feedback (i.e. praise) led to higher spontaneous movement synchrony^18^. Its importance, however, is probably most noticeable when performing music or dance. Without our ability to time, specifically to synchronize our movements to the same rhythm the production of music or dance would be hard to imagine^19^. Both music and dance have been understood as fundamental parts of society and culture, bringing people together and strengthening social bonds^19^. Due to movement synchronization’s strong embedding in our social life, investigating a connection to physiological synchrony, potentially even elevating it, is worthwhile studying. A few studies already demonstrated that synchronization of motor activity, specifically during joint vocalization^20,21^, drumming together^11^ or by mirroring movement^22^, provoked an increased synchronization of the heart rate. However, the factors that provoke this cardiac PS yet remain to be seen.

Generally, although the phenomenon of cardiac PS has been recorded in a variety of experimental settings and linked to different social or environmental causes^8^, there are still plenty of uncertainties when it comes to the question of what actually drives the increased alignment of two or more heart rate time series. Specifically for cardiac PS during joint motor activity, only speculations have been made: equal metabolic demands due to shared behaviours^22^ or simultaneously experienced extrinsic triggers due to the task condition^23^, as well as psychophysiological processes initiated by interpersonal behavioural signals (i.e. facial cues)^24^ or by sharing the same mental intention^11^. For instance, during synchrony of movement, synchronizing metabolic demands or provoking simultaneously experienced states of arousal and relaxation heart rate fluctuations might align^22,23^. While to our knowledge there is no study that directly analysed the link between interpersonal finger tapping synchronization and cardiac PS, there is however evidence of intrapersonal synchronization of heart rate fluctuations and repetitive movement, i.e. during walking and running^25^. Thus, in tasks that aim for movement synchronization, this may translate into PS. However, heart rate regulation itself is influenced by a multitude of physiological, psychological, and environmental factors, often presenting simultaneously^26^. Thus, narrowing down changes in synchrony between two hearts to only a few, preferably one specific factor requires a more controlled systematic approach^27^. Through thorough selection of a suitable study design (i), control analysis (ii), as well as inclusion of additional parameters (iii), we aim to create a more controlled approach to rule out or quantify potential variables and evaluate their impact on cardiac PS during joint finger tapping:

For this study, we chose a finger tapping task with a baseline condition. Finger tapping is a simple motion to perform, easy to record yet allows for highly variable rhythmical patterns to synchronize to. Furthermore, it is well-studied in the field of sensorimotor synchronization^28,29^, where it was demonstrated that selectively blocking sensory modalities by which the pacing rhythm is perceived leads to different degrees of tapping synchrony^29^. Therefore, while it has been brought to attention that finger tapping tasks might not deliver an equally strong basis for emotional engagement, as compared to a joint musical task^11,30^, it however allows for investigating the impact of easily quantifiable different levels of movement synchrony on PS. This contrasts previous research where tasks revolved around only two conditions of synchronized and asynchronized task execution^11,21^, a somewhat ON/OFF approach. By producing different levels of synchrony, we suggest that a more precise investigation of the interaction between movement synchrony and cardiac PS might be possible. As in real-life dyadic interaction, rhythmicity is produced by either one or both of the participating parties, a self-paced tapping paradigm might be most realistic. Hereby the participants will alternately take on two roles: the sender, who is producing the rhythm, and the receiver, who follows it. This distinction is also important since depending on the role, movement synchronization is processed differently by the corresponding brain areas, namely the basal ganglia and the cerebellum^31^. Additionally, since cardiac PS during task execution could arise from continuously present factors, i.e. chance findings of spontaneously overlapping heart rate fluctuation^32^, or mere copresence increasing PS^33^, a baseline condition incorporating them is needed. Consequently, this allows relating changes in PS during finger tapping to other potential causes.

Control analysis in PS research revolves around various types of analysis and depending on the task performed can also be differently interpreted. The most typical control analysis in PS research is to test the null hypothesis by measuring the PS of randomly combined virtually produced pairs and comparing these results to the original pairs’ PS^8^. This enables to distinguish whether PS is pair-specific and driven by a unique component of the individual interaction or whether PS is pairwise-independent and driven by a uniformly present factor, thus narrowing down potential causes for increased PS during task execution. There is evidence for pair-specific cardiac PS in various forms of social interaction^34–36^ and, specifically, during motor synchronization tasks (i.e. drumming)^11^. Yet there is also one study that could only provide pairwise-independent cardiac PS during a joint game task^23^.

To broaden our analysis one additional physiological parameter was included: heart rate variability (HRV). HRV is a characteristic of cardiac activity, i.e. the beat-to-beat changes of the heart rate with random and non-random fluctuations^37^, where high values represent more variable rhythmical patterns while low values demonstrate more uniformity across the heart rate time series. HRV is the result of several factors modulating cardiac regulation leading to multiple fluctuations in heart rate^38^. It represents an important additional parameter, as we believe, it allows for an additional perspective on the subject. HRV has consistently been linked to both stress responses (decreasing HRV) as well as emotional regulation and social engagement (increasing HRV)^39,40^, factors that very well could drive cardiac PS^24^. In regards to detecting acute stressors, it demonstrates a higher sensitivity than resting heart rate measurements^41^. Furthermore, it has been used to help characterize an individual’s mental state according to their heart rate pattern^42^. Therefore, by incorporating HRV into our analysis we might be able to further relate psychological processes to changes in PS. In order to directly compare cardiac PS and HRV we intend to extract our results from two different frequency bands that relate to neuronal cardiac regulation: high-frequency band (HF; 0.15–0.4 Hz) representing solely parasympathetic input and low-frequency band (LF; 0.04–0.15 Hz), representing parasympathetic and sympathetic inputs combined^43^. While synchrony between two or more HRV signals has been studied^44,45^, individual frequency-domain HRV has to our knowledge not been used to further characterize changes in cardiac PS.

With this more systematic approach, we aim to investigate potential drivers of cardiac PS by analysing cardiac PS in specific frequency bands during a finger tapping synchronization task between two cooperating individuals. By creating different levels of self-paced movement synchrony, we seek to study if PS relates to the act of synchronized motion or other factors. Through further characterization via control analysis and inclusion of HRV we aim to narrow down what effectually drives PS. In the supplementary materials, there will be additional analysis focusing on how a self-paced approach to tapping synchronization affects tapping accuracy according to different sensory communication modalities. With our study, we hope to gain further insight into the underlying mechanism of PS during synchronized finger tapping and exemplify a more precise approach to performing PS research.

## Results

Twenty healthy dyads (mean age 23.7 years, age range 18–35 years, 19 males, 21 females, right-handed) were measured and 40 sessions were recorded. Due to insufficient data quality, 2 dyads were fully excluded (4 sessions) and half the sessions of 2 other dyads (2 sessions) were also excluded from data analysis, i.e. the data of 18 dyads and 34 sessions were analyzed.

### Tapping data

**Figure 1:**
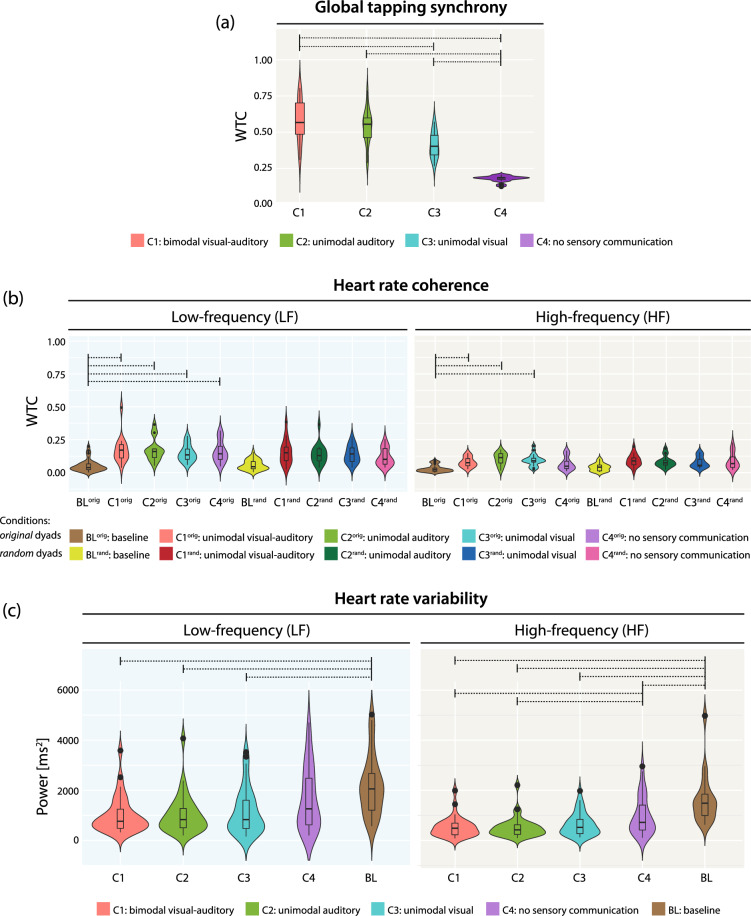
(**a**) Global finger tapping synchrony according to condition. Comparisons marked with dotted bars express significant differences (*p* < 0.05). (**b**) Interindividual heart rate coherence of original and randomized dyads according to condition and baseline. High-frequency band (0.15–0.4 Hz). Low-frequency band (0.04–0.15 Hz). Comparisons marked with dotted bars express significant differences (*p* < 0.05). In this figure, only the significant comparisons relevant to our discussion have been considered. To view all comparisons, see Supplementary Materials. (**c**) Intraindividual heart rate variability according to condition and baseline. High-frequency domain (0.15–0.4 Hz). Low-frequency domain (0.04–0.15 Hz). Comparisons marked with dotted bars express significant differences (*p* < 0.05).

By evaluating the effect of blocking sensory communication channels on global tapping synchrony (Fig. 1a), we found no statistically significant difference in tapping synchrony between condition 1 (C1; no channel blocked) and condition 2 (C2, visual channel blocked) and between C2 and condition 3 (C3, auditory channel blocked). Condition 1 demonstrated significantly higher synchrony than condition 3 (*p* < 0.05). In condition 4 (C4, no sensory communication), tapping was significantly less synchronized than in the other three conditions (*p* < 0.001, except for C3–C4 *p* = 0.004).

Average tapping speeds were consistent across all four conditions. None of the dyads’ tapping frequencies exceeded our tapping speed limits (Table 1).

**Table 1:** Tapping frequencies across conditions (C1–4). No significant difference between average tapping frequencies was found.

	Tapping frequency (Hz)			
	C1	C2	C3	C4
Mean	1.812	1.946	1.691	1.725
SD	0.360	0.448	0.368	0.511
Variance	0.130	0.201	0.135	0.261
Minimum	1.400	1.430	1.105	0.673
Maximum	2.593	3.142	2.404	2.981

Further analysis and interpretation of tapping data are provided in the Supplementary Materials (Fig. S1).

### Heart rate coherence

We quantified cardiac PS by the wavelet transformation coherence index in the two above-mentioned frequency bands across conditions and baseline (BL) of both original and random pairings (Fig. 1b). This analytical product we termed heart rate coherence. The results of the original and random dyad’s analysis were consistent in any of the frequency bands, with a significant increase in HR coherence from BL to all conditions in the low-frequency (LF, *p* < 0.001, *ε*^2^ = 0.37) and in the high-frequency band (HF, *p* < 0.001, *ε*^2^ = 0.29). Only between BL and the control condition (C4) in the HF band, no significant difference remained after Holm-correction (*p* = 0.115). Following inter-condition comparison, no notable differences were demonstrated between any of the conditions in both frequency bands.

During control analysis, no difference between original and randomized dyads’ PS was found, as neither BL (BL^orig^–BL^rand^) nor condition comparisons (C1^orig^–C1^rand^; C2^orig^–C2^rand^; etc.) expressed any significant difference.

Phase angles between senders’ and receivers’ time series are displayed in Table 2. No significant difference across all conditions of original and control analysis in LF and HF were found.

**Table 2:** Phase angle of heart rate coherence according to condition (C1–4) of original (orig) and control analysis (rand). No statistically significant differences across conditions.

		Phase angle (π)							
		C1_orig	C2_orig	C3_orig	C4_orig	C1_rand	C2_rand	C3_rand	C4_rand
HF	Mean	0.088	− 0.203	− 0.030	0.153	0.160	0.173	− 0.005	0.120
	SD	0.319	0.345	0.373	0.285	0.296	0.344	0.321	0.262
LF	Mean	− 0.071	− 0.173	0.038	0.225	0.229	0.210	0.046	0.139
	SD	0.420	0.725	0.453	0.538	0.167	0.156	0.455	0.584

More detailed statistics including p-values before Holm-correction and further comparisons are provided in Supplementary Materials (Tables S3–5).

### Heart rate variability

As depicted in Fig. 1c, almost consistent differences between averaged BL and conditions of both HRV HF (*p* < 0.001, *ε*^2^ = 0.30) and LF (*p* < 0.001, *ε*^2^ = 0.15) were found. Post-hoc Dunn test results showed significantly higher values in BL compared to the condition values (*p* < 0.001; except for the LF band’s BL—C4, *p* = 0.104). In inter-condition comparison, HRV LF showed equally low results across all conditions. HRV HF, however, while demonstrating no significant difference between C1, C2, and C3 power spectral density, was significantly lower in both C1 and C2 as compared to C4 (*p* < 0.001). A significant difference between visual (C3) and no sensory communication (C4) was not observed (*p* = 0.284).

## Discussion

The study aims to investigate cardiac PS during a self-paced motor entrainment task. The analysis focused on how different degrees of tapping synchrony during a cooperative finger tapping task affected the individuals' heart rate regulation and interpersonal synchronization of heart rate time series. In search of a link between movement synchronization and heart rate regulation, several mechanisms were considered. Our results demonstrate that increased cardiac PS was elicited by simultaneous tapping, as shown by the changes from BL to all the conditions in both frequency bands (except for BL^orig^ to C4^orig^ of the HF band). Consequently, the tapping task increased cardiac synchrony. Yet, the control analysis showed no difference in HR coherence between correctly paired and randomized dyads. We could, therefore, not attribute the rise in PS to a dyad-specific interaction.

The results of the control analysis indicate a uniformly present mediator for PS during the synchronization task. Strang et al.^23^ encountered similar results by random pairing for a Tetris-like video game, where one participant controlled the rotation of the bricks while the other controlled the lateral positioning. Contrary to their initial hypothesis, they also did not find any PS unique to the original dyads. They explained this by overarching aspects of team membership and task demands^23^. To our knowledge, a concrete explanation for this dyad-unspecific PS is missing. Adapted from Palumbo et al.^8^ we, therefore, formulate three hypothetical explanations: (i) a chance finding, (ii) quasi-simultaneous conditional demands, and (iii) equally shared psychophysiological mode.

PS generated by *chance* may appear by random overlaps between the two heart rate time series^32^. As chance findings appear randomly, the resulting PS should be ubiquitously present^23^. This would explain the similar cardiac PS of correctly and randomly paired dyads, but not the increase in heart rate coherence from baseline to task execution. Therefore, we exclude chance as an explanation.

Exposure to *quasi-simultaneous conditional demands* may provoke processes synchronizing cardiac regulation due to similar surges and drops in metabolic demands^22^, similar external/environmental stressors^23^, or alignment of respiration^20^. During a synchronization task this strongly relates to task performance, i.e. synchrony. One example is singing in a choir. By the synchronized muscle contraction necessary for joint vocalization, similar metabolic demands are created. Further, due to the similar breathing intervals determined by the song’s structure, an alignment of respiratory sinus arrhythmia occurs. Thus, singing in a choir will produce increased PS across all members^20^, and performing a control analysis by randomly pairing this data with data from another choir singing the same song is expected to produce similar cardiac PS. It must be noted, however, that breathing synchronization can be both a result of task constraint as well as interpersonal processes^46^ and, moreover, will not always impact cardiac PS^10^. In this study, quasi-simultaneous conditional demands could account for the changes from BL to conditions but fail to explain the similar PS of both original and randomized dyads across all conditions. In the original dyads’ analysis, HR coherence and tapping synchrony did not correlate. This can be seen in C4^orig^, where tapping synchrony was significantly lower than in all other conditions, yet HR coherence remained similarly elevated. Since lower tapping synchrony indicates less similar task execution between the senders and receivers, the almost unchanged HR coherence in C4^orig^ can, therefore, not be regarded as a consequence of quasi-simultaneous conditional demands. Previous research demonstrated that synchronization of heart rate to movement positively correlates with increased metabolic demand and more consistently occurs during more intense muscle activity^47,48^. This supposedly embodies a loss of regulatory complexity due to very high metabolic demands overruling other more nuanced regulatory pathways in order to maintain optimal blood flow to the working musculature^49^. Thus, it comes as no surprise that a rather light movement task such as finger tapping might not be a strong enough driver for the coupling of movement and heart rate. In addition, the tapping synchrony between original and control analysis was different, i.e. the self-paced tapping task was highly variable between dyads (see Supplementary Materials Sect. S1). This indicates that randomly paired dyads did not experience any similarly timed stimuli. Yet, the HR coherences of original and randomly paired dyads were similar. Therefore, quasi-simultaneous tasks are an unfitting explanation for these results.

Lastly, equally shared *psychophysiological modes* are shared states of mind that similarly modulate autonomic regulation in a way that facilitates or even mediates PS. These alterations of the state of mind might be provoked either by general interpersonal stimuli, such as co-presence, or by a task-related stimulus, that all participating individuals experience. According to the findings of McCraty et al.^42^, these modes appear to specifically alter autonomic HR regulation, creating mode-corresponding fluctuation patterns. Consequently, if all individuals entered the same state of mind, the adopted more uniform HR time series could well lead to similar HR coherence across original and randomized dyads, independent of task performance. Gordon et al.^11^ came to a similar interpretation after they found increased cardiac PS from baseline to task execution but no difference between synchronous and asynchronous drumming. They could not relate cardiac PS to the actual act of drumming but to the intention to do so. Having an intention is regarded as a mental state^50^. It must be mentioned, however, that in contrast to our study their results showed significantly higher PS in the original dyads than in the virtual dyads of the control analysis. The mere co-presence does not account for the increase in cardiac PS from BL to task execution, since in BL there was co-presence as in other conditions. This rules out co-presence as a factor. Yet, task-related mode alterations could very well be a fitting explanation for our findings. In contrast to intention^11^ we believe it might relate to another mental state. To perform a synchronization task, mental effort is necessary. HRV expresses an inverse relationship to mental effort^51^. Accordingly, our results show a significant decrease in HRV HF and LF during task execution compared to BL. Thus, while changing from relaxing at BL to performing a task, participants enter a different psychophysiological mode of higher mental effort. Mental effort is a term, that, depending on the challenge, relates to different brain functions (e.g. sustained attention, sensory processing of rhythmicality, production of accurately timed motor programs, etc.)^52^. For our experiment, it is not possible to pinpoint the psychophysiological mode to a specific brain function. In their attempt to characterize different psychophysiological modes by analyzing patterns in HR time series and HRV, McCraty et al.^42^ obtained relatable results. They found that the mode of “mental focus” provoked a less variable HR fluctuation pattern while maintaining a relatively constant HR^42^; see Appendix A. In accordance, our results in HRV confirm this loss of variability from BL to task execution. We hypothesize that participants entered into the psychophysiological mode related to mental effort, which created more uniform, less variable HR fluctuation patterns. These more congruent HR patterns in return enabled the recording of equally elevated cardiac PS during task execution in original and control analysis. Whether the more homogeneous HR patterns truly temporally aligned even across the randomized dyads or whether the higher HR coherence occurred due to less variable, more similar fluctuations tending to randomly overlap more often remains to be seen. Judging from the rather large standard deviations across phase angles of all conditions, that display multiple receiver's time series to both lead (negative phase angle) as well as follow (positive phase angle) the senders time series, we however think the latter is more likely. However, for C4 deviating results were recorded: HRV LF was not significantly different from BL, and HRV HF demonstrated significantly lower values than for C1 and C2. This indicates that during C4 mental effort decreased since no communication was possible and participants just tapped for themselves. Consequently, while some individuals might still have remained in the same psychophysiological mode others might have entered into a more effortless state of mind. This inhomogeneity might be the reason for the insignificant difference between BL^orig^ and C4^orig^ in the HR coherence HF band. This in turn suggests that during the psychophysiological mode of mental effort, HF components deviate more easily from their typical HR fluctuation pattern to changes in mental effort while LF components remain more consistent. It must be noted, however, that before Holm-correction, comparisons of both HRV and HR coherence data showed consistently significant differences between BL and all four conditions (see Supplementary Materials Tables S3–8). Therefore, it is plausible, that even during C4 a more uniform fluctuation pattern prevailed, which produced higher cardiac PS than the BL. To sum up, an equally shared psychophysiological mode seems a valid explanation for our results.

Even though our study did not provide evidence for singular interpersonal PS, other researchers showed significantly higher PS in original than in random pairings^11,34–36^. As PS is associated with emotional^53,54^, attachment and coregulatory processes^24^, this discrepancy to our study could be explained by the low emotional and/or social engagement involved in our tapping task. This might also distinguish our findings from the above-mentioned study of Gordon et al.^11^.

This study has some limitations. Firstly, since we did not randomize the order of the conditions, our findings might not only relate to partial or complete sensory channel deprivation. There could be a learning effect. Learning could have increased tapping synchronization. Specifically, the similarity in tapping performance during C1, C2, and C3 could be related to the learning process: the potential loss in tapping synchrony would have been compensated by learning to perform the task more efficiently. This could also explain the discrepancy of our results from other research^29^. Since we encouraged the participants to choose their own tapping rhythms and continuously alter them, each condition consisted of its own unique sender-specific set of rhythms, potentially complicating any learning process. Furthermore, a more recent study suggests a lack of learning effects in finger tapping tasks when measurements are performed within a day and to only manifest after a longer period of time (i.e. 1 week)^55^. Consequently, differences in tapping synchrony between C1–C2, and C3–C4 respectively might not be affected. There is, however, the possibility that learning positively affected performance during C3 and C4, thus diminishing an otherwise significant difference between C2 (auditory) and C3 (visual). Secondly, facing each other during conditions with visual contact could have triggered a decrease in performance due to attentional disturbances of social interaction^56^. But the task execution forces the gaze to be focused on the partner’s hands rather than his/her face. Therefore, results relating to direct eye contact do not apply to our tapping tasks. Lastly, during our BL, subjects were allowed to socially interact such as talking BL. This may be a factor limiting the comparability of our results to other research, where strict conditions are imposed during BL (e.g. sitting with knees at a 90° angle, both feet flat on the floor, hands on thighs, and eyes closed^40^). Free talking was demonstrated to affect HRV by lowering the breathing frequency which provokes respiratory sinus arrhythmia to contribute more to the LF range of HRV, although it did not significantly alter HRV and HF from spontaneous breathing conditions^57^. Yet, a more recent meta-analysis found no difference between HRV and HF in resting state (e.g. sitting still with closed eyes) and neutrally or positively connotated social interaction^58^. This is also true for our experiment, where we never observed any antagonism. Regarding HR coherence, we would expect social interaction to increase HR synchronization^59^, potentially diminishing the difference between BL and task conditions. Yet even under this setting baseline HR coherence was different from task execution and thus the effect of talking was irrelevant to our results.

In conclusion, we demonstrated that increased cardiac PS during a self-paced interpersonal tapping synchronization task was not unique to the original pairings but would also emerge from randomly combined pairings as well. We have reason to believe that this universal surge in PS resulted from a task dependent mechanism participants entering into a similar psychophysiological mode. The mode, induced by mental effort, adapted their HR regulation to more uniform, less variable rhythms, thus prompting more overlaps between individuals' HR time series. Our approach of combining task execution with BL and control conditions and performing post hoc control analysis by randomization enabled us to show that HR coherence occurred generally, i.e. also dyad-unspecifically. Lastly, we suggest that analysing patterns of HR time series according to psychological modes, especially combined with HRV analysis, might be an interesting new approach to consider in physiological synchrony research. While the phenomenon of cardiac PS, specifically its genesis and function still is a matter of debate, we hope these insights might further its understanding.

## Methods

### Subjects

The study was conducted at the Biomedical Optics Research Laboratory, University Hospital in Zurich, Switzerland. Twenty dyads of volunteers were recruited. Age and gender were recorded. Participants had to be right-handed and healthy with no prior medical conditions.

### Instrumentation

HR and RR-Intervals were recorded using a 3-channel continuous non-invasive ECG (SOMNOtouch™ NIBP, SOMNO Medics, Germany). The electrodes were placed in a modified Einthoven configuration on the chest. Tapping was recorded with two keyboards (HP™ Classic Wired Keyboard) connected to a Raspberry Pi (Model 1). For observing tapping requirements, the default stopwatch app on an iPhone (Model 5) was used. Additionally, as part of a related research project the participant’s scalp electric potential was recorded using 32 active EEG (ActiCap32 Setup, Brain Products GmbH, Germany) and oxygen metabolism and perfusion were recorded using an fNIRS device (NIRSport 1, NIRx Medizintechnik GmbH, Germany). However, since they did not contribute to the findings of this study, they will not be mentioned further.

### Study protocol

First, all participants provided written informed consent. All participants were advised to perform no excessive physical activity^60^ and avoid caffeine consumption^61^ for 12 h before the experiment and were suggested to use the toilet directly before the start of the measurement^62^. After receiving the consent, each dyad sat on opposite sides of a long table facing each other and all measurement instruments were set up, turned on, and started recording. At the end of the installation process, participants were instructed to place their right index finger on the recording button of the keyboard (Enter or Space, respectively). Pressing the button created a clicking sound.

**Figure 2:**
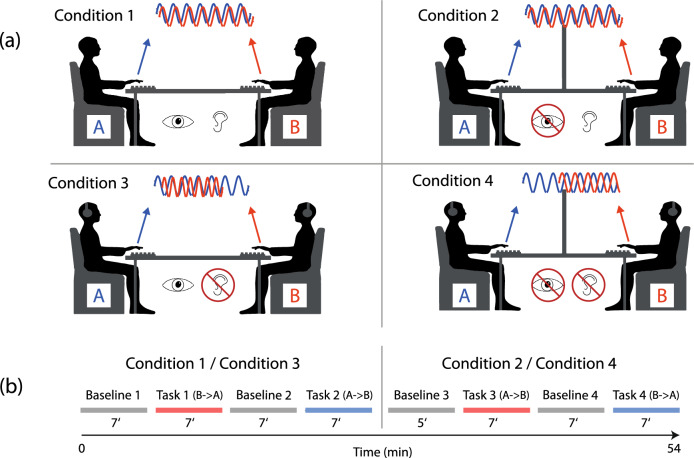
Visualization of the (**a**) four task conditions and the (**b**) experimental paradigm for participants A and B.

The protocol included two 54-min sessions on two different days. It was intended to perform the two sessions at the same time of day^63^. Four different conditions were examined in the following order: (condition 1 C1) bimodal visual-auditory, (condition 2 C2) unimodal auditory, (condition 3 C3) unimodal visual, and (condition 4 C4) no sensory communication (Fig. 2a). C4 was considered the control condition. Each session was made up of two different sensory communication conditions, each consisting of two tasks and two baselines (BL) following a strict paradigm: (i) 7 min BL 1, (ii) 7 min task 1, (iii) 7 min BL 2, (iv) 7 min task 2, (v) 5 min BL 3, (vi) 7 min task 3, (vii) 7 min BL 4 (viii) 7 min task 4 (Fig. 2b).

During the BL, the participants were instructed to remain seated but were allowed to engage with each other and were allowed to drink. During the 7-min tasks, the participants were instructed to continuously tap together with the intention of maximal synchronization of their tapping rhythm. One participant was considered the “Sender” creating the rhythm and the other was considered the “Receiver” trying to follow the rhythm and synchronize with it. For every other task, the two roles were exchanged. Therefore, in Task 1 and Task 4, one participant assumed the role of sender and during Task 2 and Task 3 the roles were reversed. While the “receiver” aimed to follow and synchronize with the produced rhythm in a 1:1 fashion, the “sender” was instructed to create and alter any rhythm he deemed followable, isochronous and non-isochronous alike, with the only two requirements being the range of tapping speed (max. < 5 taps/s^64^; min. > 0.5 taps/s^65^) and the maintenance of the same rhythm for at least 40 s once perceived synchronization had occurred, before changing to an alternative rhythm. To assure adherence to the requirements an examinator checked tapping speeds and maintenance of the same rhythm with a stopwatch and gave verbal feedback accordingly. To apply the conditions correctly, we adjusted the laboratory environment accordingly: (i) In the first session, after performing two tasks with bimodal input, the visual contact was blocked by placing a big styrofoam board between the two participants and only the acoustic button clicking remained as reference. (ii) For the first two tasks of the second session, auditory input was blocked through the insertion of earplugs in both participants' ears, and (iii) for the final two tasks both the earplugs and the styrofoam board were used to abolish any sensory input between the pairing. To prevent any transmission of tactile information (e.g. touching each under the table or stomping on the floor, creating vibrations), the two participants were seated 2 m apart, and we asked to not tap along with the other hand on the table or with their feet on the floor.

The study protocol was approved by the ethics committee of the County of Zurich (KEK-ZH-Nr. E50/2002) in accordance with relevant guidelines and regulations.

### Data analysis

Signal processing was performed in Matlab (The MathWorks, Inc., Natick, MA, USA, version R2020b) and statistical analysis in R (version 3.6.2).

### Tapping data

A tapping coherence index for each dyad and each condition was calculated. To this end, the keystroke sequence recorded in the log files belonging to each performer was transformed into sinusoidal signals of variable period proportional to the time of two consecutive digits. Next, the synchrony was determined using the wavelet transform coherence^66,67^ excluding the coherence values outside the cone of influence. We calculated the mean of the coherence over two different frequency bands: (i) bimodally processable [0.5–2.17 Hz] and (ii) acoustically processable [2.17–7.0 Hz]. These frequency ranges are based on established limits of motor entrainment, i.e., the slowest accurately reproducible frequency of 0.5 Hz and the highest visually processable one of 2.17 Hz, and the highest one at which the effector can move (7 Hz). Auditory frequency processing (9–10 Hz) exceeds the maximal speed of motion^29^. We then averaged the wavelet transform coherence over time obtaining a global index for each condition and frequency range. The statistical significance was assessed using the Kruskal–Wallis rank-sum test among the four conditions at a significance level of *p* < 0.05. The effect size of the Kruskal–Wallis rank-sum test was computed as the eta squared based on the H-statistic (η^2^_p_(H)). The Dunn test was evaluated to calculate the pairwise comparisons between group levels with the Holm corrections for multiple testing.

The average, as well as minimum and maximum of the tapping rates recorded during the 7 min tasks for each condition and dyad were calculated. To assess any differences among the different conditions the Kruskal–Wallis test was performed.

### Heart rate coherence

The HR and RR-interval time series were first extracted using the DOMINO light software (SOMNOtouch™ software). Then, the participants’ HR signals were smoothed by the Savitzky–Golay filter (polynomial order 3 and frame length 61). To evaluate the HR synchrony between the dyads, the wavelet transform coherence method was applied. This method allows analyzing the coherence between two-time series as a function of both frequency and time. The coherence can assume values in the range between [0–1], where 1 means that the two signals are highly correlated, and 0 indicates that there is no correlation in a certain instant of time and at a specific frequency. The mean over two frequency bands was evaluated: (i) low-frequency [0.04–0.15 Hz], and (ii) high-frequency [0.15–0.40 Hz]. After that, the mean coherence was calculated for each condition. The four BL coherence values were averaged obtaining one overall BL value per dyad (referred to as ‘BL’). To assess whether the coherence of HR was statistically different between the conditions and BL, a Kruskal–Wallis rank-sum test was performed as well as the subsequent Dunn test as post hoc analysis and Holm corrections for multiple testing.

We performed an additional analysis considering the phase angle in the heart coherence analysis. The analysis was performed in the two frequency bands (LF and HF) among the eight conditions (obtained by real and random pairs). The phase angle was obtained from the output of the wavelet coherence function in MATLAB (wavelet cross spectrum) and then calculating the median along the two frequency bands. Receivers’ time series were analysed with respect to senders’ time series. For the comparison of the conditions of the original and random pairs, a Kruskal–Wallis test was performed as well as the subsequent Dunn test as post hoc analysis and Holm corrections for multiple testing.

### Heart rate variability

The RR time series of each participant was preprocessed in MATLAB as specified by Ref.^68^. In addition to physiological artefacts removal (ectopic beats and arrhythmic events), the linear trends were also removed. After that, using Welch’s method^69,70^ in MATLAB, the power spectral density was calculated for the preprocessed RR interval traces, considering the HRV low- and high-frequency bands (HRV LF: [0.04–0.15 Hz], HRV HF: [0.15–0.4 Hz]). The HRV LF and HF were evaluated for each task and BL. The previous analysis was performed with custom MATLAB functions. Then, the Kruskal–Wallis rank-sum test was performed as well as the subsequent Dunn test as post hoc analysis and Holm corrections for multiple testing.

### Control analysis

The wavelet transform coherence for the HR signals for 60 random dyads was calculated as control analysis. The control wavelet transform coherence values were averaged over the conditions (including BLs) and the two frequency bands (LF and HF). The Wilcoxon rank-sum test was applied to compare the distribution of the true HR wavelet transform coherence values with the spurious ones. The same procedure was performed for the tapping coherence analysis as well.

## Supplementary Information

Supplementary Information.

## Data Availability

The datasets generated and analyzed during the current study are available from the corresponding author upon reasonable request.
