# Supplementary material for: How our hearts beat together: a study on physiological synchronization based on a self-paced joint motor task
Source: Sci Rep. 2023 Jul 25;13:11987. doi: 10.1038/s41598-023-39083-9 (PMC10368740; doi:10.1038/s41598-023-39083-9)
Supplement: Supplementary file 1 — Supplementary Information. [file 41598_2023_39083_MOESM1_ESM.pdf]

Supplementary Materials for

How our hearts beat together – A study on physiological synchronization based on a self-paced joint motor task

Stephan Flory[1,*,†], Sabino Guglielmini[1,†], Felix Scholkmann[1,2,3], Valentine Marcar[1,4] and Martin Wolf[1]

[1] Biomedical Optics Research Laboratory, Department of Neonatology, University Hospital Zurich, University of Zurich, Zurich, Switzerland
[2] Neurophotonics and Biosignal Processing Research Group, Department of Neonatology, University Hospital Zurich, University of Zurich, Switzerland
[3] Institute of Complementary and Integrative Medicine, University of Bern, Bern, Switzerland
[4] Comprehensive Cancer Center Zürich, University Hospital Zürich, Zürich, Switzerland

[*]Corresponding author: Stephan.Flory@usz.ch
[†]These authors contributed equally to this work

Main Analysis

Table S1 Kruskal-Wallis rank sum test results of the global tapping synchrony among the four original conditions (obtained by real pairs). In the table are listed the degree of freedom (*df*), chi-squared (χ^2), the p-values (*p*), and the eta-squared (ε^2) with the respective magnitude. The significant *p*-values are marked in bold typeface.

Signal	*df*	χ^2	*p*	ε^2	Magnitude
**Global tapping synchrony**	3	81.99	**< 0.001**	0.597	large

Table S2: Post-hoc Dunn test results of the global tapping synchrony among the four original conditions (obtained by real pairs). In the table are listed the *z*-test statistic (*Z*), the *p*-values (*p*), and the Holm-adjusted *p*-values (*p*.adj). All the significant adjusted *p*-values are marked in bold typeface.

Comparison	Z	*p*	*p.adj*
C1 - C2	0.45	0.655	0.655
C1 - C3	2.57	0.010	**0.031**
C2 - C3	2.14	0.033	0.065
C1 - C4	5.94	< 0.001	**< 0.001**
C2 - C4	5.51	< 0.001	**< 0.001**
C3 - C4	3.28	0.001	**0.004**

Table S3: Kruskal-Wallis rank sum test results of the HR coherence over the two frequency bands (LF and HF) among the eight conditions and the two baselines (obtained by real and random pairs). In the table are listed the degree of freedom (*df*), chi-squared (χ^2), the *p*-values (*p*), and the eta-squared (ε^2) with the respective magnitude. A single asterisk indicates $p < 0.05$ after the FDR correction.

Range	*df*	χ^2	*p*	ε^2	Magnitude
**HR coherence LF [0.04-0.15 Hz]**	9	79.25	**< 0.001** *	0.37	large
**HR coherence HF [0.15-0.4 Hz]**	9	64.87	**< 0.001** *	0.29	large

*** Significant *p*-values after the FDR correction**

Table S4: Post-hoc Dunn test results of the HR coherence in the LF band among the eight conditions and two baselines (obtained by real and random pairs). In the table are listed the *z*-test statistic (*Z*), the *p*-values (*p*), and the Holm-adjusted *p*-values (*p*.adj). All the significant adjusted *p*-values are marked in bold typeface.

**Comparison**	**Z**	***p***	***p.adj***
BL^{orig} - $C1^{orig\,\#}$	-5.09	< 0.001	**< 0.001**
BL^{orig} - $C2^{orig\,\#}$	-5.14	< 0.001	**< 0.001**
BL^{orig} - $C3^{orig\,\#}$	-4.76	< 0.001	**< 0.001**
BL^{orig} - $C4^{orig\,\#}$	-4.98	< 0.001	**< 0.001**
$C1^{orig}$ - $C2^{orig\,\#}$	-0.04	0.970	0.970
$C1^{orig}$ - $C3^{orig\,\#}$	0.55	0.583	1.000
$C1^{orig}$ - $C4^{orig\,\#}$	0.37	0.714	1.000
$C2^{orig}$ - $C3^{orig\,\#}$	0.59	0.556	1.000
$C2^{orig}$ - $C4^{orig\,\#}$	0.41	0.685	1.000
$C3^{orig}$ - $C4^{orig\,\#}$	-0.19	0.848	1.000
BL^{orig} - $BL^{rand\,\#}$	-0.47	0.638	1.000
$C1^{orig}$ - $C1^{rand\,\#}$	0.66	0.511	1.000
$C2^{orig}$ - $C2^{rand\,\#}$	0.93	0.351	1.000
$C3^{orig}$ - $C3^{rand\,\#}$	0.34	0.737	1.000
$C4^{orig}$ - $C4^{rand\,\#}$	1.40	0.162	1.000
**Additional Comparisons**			
BL^{rand} - $C1^{orig}$	-4.72	< 0.001	**< 0.001**
BL^{orig} - $C1^{rand}$	-4.43	< 0.001	**< 0.001**
BL^{rand} - $C1^{rand}$	-4.05	< 0.001	**0.002**
BL^{rand} - $C2^{orig}$	-4.77	< 0.001	**< 0.001**
$C1^{rand}$ - $C2^{orig}$	-0.70	0.487	1.000

BL^{orig} – $C2^{rand}$	-4.15	< 0.001	**0.001**
BL^{rand} - $C2^{rand}$	-3.77	< 0.001	**0.005**
$C1^{orig}$ - $C2^{rand}$	0.89	0.372	1.000
$C1^{rand}$ - $C2^{rand}$	0.24	0.810	1.000
BL^{rand} - $C3^{orig}$	-4.36	< 0.001	**< 0.001**
$C1^{rand}$ - $C3^{orig}$	-0.13	0.898	1.000
$C2^{rand}$ - $C3^{orig}$	-0.38	0.707	1.000
BL^{orig} - $C3^{rand}$	-4.29	< 0.001	**0.001**
BL^{rand} - $C3^{rand}$	-3.90	< 0.001	**0.003**
$C1^{orig}$ - $C3^{rand}$	0.86	0.388	1.000
$C1^{rand}$ - $C3^{rand}$	0.20	0.842	1.000
$C2^{orig}$ - $C3^{rand}$	0.90	0.367	1.000
$C2^{rand}$ - $C3^{rand}$	-0.04	0.965	1.000
BL^{rand} - $C4^{orig}$	-4.58	< 0.001	**< 0.001**
$C1^{rand}$ - $C4^{orig}$	-0.31	0.753	1.000
$C2^{rand}$ - $C4^{orig}$	-0.56	0.574	1.000
$C3^{rand}$ - $C4^{orig}$	-0.53	0.600	1.000
BL^{orig} - $C4^{rand}$	-3.30	0.001	**0.030**
BL^{rand} - $C4^{rand}$	-2.91	0.004	0.108
$C1^{orig}$ - $C4^{rand}$	1.70	0.090	1.000
$C1^{rand}$ - $C4^{rand}$	1.05	0.294	1.000
$C2^{orig}$ - $C4^{rand}$	1.74	0.082	1.000
$C2^{rand}$ - $C4^{rand}$	0.80	0.421	1.000
$C3^{orig}$ - $C4^{rand}$	1.21	0.226	1.000
$C3^{rand}$ - $C4^{rand}$	0.86	0.389	1.000

# Comparisons considered for discussion

Table S5: Post-hoc Dunn test results of the HR coherence in the HF band among the eight conditions and two baselines (obtained by real and random pairs). In the table are listed the *z*-test statistic (*Z*), the *p*-values (*p*), and the Holm-adjusted *p*-values (*p*.adj). All the significant adjusted *p*-values are marked in bold typeface.

Comparison	Z	*p*	*p.adj*
BL^{orig} - $C1^{orig}$ #	-3.96	< 0.001	**0.003**
BL^{orig} - $C2^{orig}$ #	-5.18	< 0.001	**< 0.001**
BL^{orig} - $C3^{orig}$ #	-5.20	< 0.001	**< 0.001**
BL^{orig} - $C4^{orig}$ #	-2.90	0.004	0.115
$C1^{orig}$ - $C2^{orig}$ #	-1.05	0.296	1.000
$C1^{orig}$ - $C3^{orig}$ #	-0.83	0.406	1.000
$C1^{orig}$ - $C4^{orig}$ #	1.09	0.274	1.000
$C2^{orig}$ - $C3^{orig}$ #	0.26	0.794	1.000
$C2^{orig}$ - $C4^{orig}$ #	2.19	0.029	0.866
$C3^{orig}$ - $C4^{orig}$ #	2.02	0.044	1.000
BL^{orig} - BL^{rand} #	-1.24	0.217	1.000
$C1^{orig}$ - $C1^{rand}$ #	-0.59	0.558	1.000
$C2^{orig}$ - $C2^{rand}$ #	0.90	0.369	1.000
$C3^{orig}$ - $C3^{rand}$ #	1.02	0.307	1.000
$C4^{orig}$ - $C4^{rand}$ #	-1.04	0.299	1.000
**Additional Comparisons**			
BL^{rand} - $C1^{orig}$	-2.98	0.003	0.095
BL^{orig} - $C1^{rand}$	-4.74	< 0.001	**< 0.001**
BL^{rand} - $C1^{rand}$	-3.74	0.000	**0.007**
BL^{rand} - $C2^{orig}$	-4.21	< 0.001	**0.001**
$C1^{rand}$ - $C2^{orig}$	-0.48	0.634	1.000
BL^{orig} - $C2^{rand}$	-4.24	< 0.001	**0.001**
BL^{rand} - $C2^{rand}$	-3.24	< 0.001	**0.042**
$C1^{orig}$ - $C2^{rand}$	-0.16	0.870	1.000
$C1^{rand}$ - $C2^{rand}$	0.43	0.668	1.000
BL^{rand} - $C3^{orig}$	-4.16	< 0.001	**0.001**
$C1^{rand}$ - $C3^{orig}$	-0.23	0.816	1.000
$C2^{rand}$ - $C3^{orig}$	-0.67	0.500	1.000
BL^{orig} - $C3^{rand}$	-3.94	< 0.001	**0.003**
BL^{rand} - $C3^{rand}$	-2.93	0.003	0.110

$C1^{orig}$ - $C3^{rand}$	0.15	0.877	1.000
$C1^{rand}$ - $C3^{rand}$	0.76	0.447	1.000
$C2^{orig}$ - $C3^{rand}$	1.23	0.218	1.000
$C2^{rand}$ - $C3^{rand}$	0.33	0.745	1.000
BL^{rand} - $C4^{orig}$	-1.86	0.062	1.000
$C1^{rand}$ - $C4^{orig}$	1.73	0.085	1.000
$C2^{rand}$ - $C4^{orig}$	1.28	0.199	1.000
$C3^{rand}$ - $C4^{orig}$	0.97	0.333	1.000
BL^{orig} - $C4^{rand}$	-4.02	< 0.001	**0.002**
BL^{rand} - $C4^{rand}$	-3.01	0.003	0.090
$C1^{orig}$ - $C4^{rand}$	0.09	0.930	1.000
$C1^{rand}$ - $C4^{rand}$	0.69	0.488	1.000
$C2^{orig}$ - $C4^{rand}$	1.16	0.244	1.000
$C2^{rand}$ - $C4^{rand}$	0.26	0.796	1.000
$C3^{orig}$ - $C4^{rand}$	0.95	0.342	1.000
$C3^{rand}$ - $C4^{rand}$	-0.07	0.945	0.945

[#] Comparisons considered for discussion

Table S6: Kruskal-Wallis rank sum test results of the HRV in frequency domain among the four conditions and the baseline. In the table are listed the degree of freedom (*df*), chi-squared (χ^2), the *p*-values (*p*), and the eta-squared (ε^2) with the respective magnitude. A single asterisk indicates $p < 0.05$ after the FDR correction.

Signal	*df*	χ^2	*p*	ε^2	Magnitude
**HRV LF [0.04-0.15 Hz]**	4	29.52	**< 0.001** *	0.15	large
**HRV HF [0.15-0.4 Hz]**	4	29.52	**< 0.001** *	0.30	large

123

*** Significant *p*-values after the FDR correction**

Table S7: Post-hoc Dunn test results of the HRV LF among the four conditions and baseline. In the table are listed the *z*-test statistic (*Z*), the *p*-values (*p*), and the Holm-adjusted *p*-values (*p*.adj). All the significant *p*-values are marked in bold typeface.

Comparison	Z	*p*	*p.adj*
BL - C1	4.36	< 0.001	**< 0.001**
BL - C2	4.31	< 0.001	**< 0.001**
C1 - C2	-0.04	0.966	0.966
BL - C3	4.21	< 0.001	**< 0.001**
C1 - C3	-0.34	0.735	1.000
C2 - C3	-0.29	0.769	1.000
BL - C4	2.43	0.015	0.104
C1 - C4	-2.03	0.042	0.252
C2 - C4	-1.99	0.047	0.233
C3 - C4	-1.78	0.075	0.301

Table S8: Post-hoc Dunn test results of the HRV HF among the four conditions and baseline. In the table are listed the *z*-test statistic (*Z*), the *p*-values (*p*), and the Holm-adjusted *p*-values (*p*.adj). All the significant *p*-values are marked in bold typeface.

Comparison	Z	*p*	*p.adj*
BL - C1	5.98	< 0.001	**< 0.001**
BL - C2	6.16	< 0.001	**< 0.001**
C1 - C2	0.17	0.863	0.863
BL - C3	5.30	< 0.001	**< 0.001**
C1 - C3	-0.92	0.356	0.712
C2 - C3	-1.10	0.270	0.810
BL - C4	3.50	< 0.001	**< 0.001**
C1 - C4	-2.64	< 0.001	**< 0.001**
C2 - C4	-2.82	< 0.001	**< 0.001**
C3 - C4	-1.80	0.071	0.284

Table S9: Kruskal-Wallis rank sum test results of the phase angle analysis over the two frequency bands (LF and HF) among the eight conditions (obtained by real and random pairs). In the table are listed the degree of freedom (*df*), chi-squared (χ^2), the *p*-values (*p*), and the eta-squared (ε^2) with the respective magnitude.

Range	*df*	χ^2	*p*	ε^2	Magnitude
**LF band [0.04-0.15 Hz]**	7	13.524	0.060	0.084	small
**HF band [0.15-0.04 Hz]**	7	15.217	**0.033**	0.133	Moderate

Table S10 Post-hoc Dunn test results of the of the phase angle analysis over the two frequency bands (LF and HF) among the eight conditions (obtained by real and random pairs).

Dunn's Post Hoc Comparisons - Condition

Comparison	z	W_i	W_j	p	p_{bonf}	p_{holm}
C1_orig - C1_rand	-0.791	62.438	72.813	0.429	1.000	1.000
C1_orig - C2_orig	1.720	62.438	39.875	0.085	1.000	1.000
C1_orig - C2_rand	-1.034	62.438	76.000	0.301	1.000	1.000
C1_orig - C3_orig	0.743	62.438	52.688	0.457	1.000	1.000
C1_orig - C3_rand	0.329	62.438	58.125	0.742	1.000	1.000
C1_orig - C4_orig	-1.196	62.438	78.125	0.232	1.000	1.000
C1_orig - C4_rand	-1.029	62.438	75.938	0.303	1.000	1.000
C1_rand - C2_orig	2.511	72.813	39.875	0.012	0.337	0.301

Dunn's Post Hoc Comparisons - Condition

Comparison	z	W_i	W_j	p	p_{bonf}	p_{holm}
C1_rand - C2_rand	-0.243	72.813	76.000	0.808	1.000	1.000
C1_rand - C3_orig	1.535	72.813	52.688	0.125	1.000	1.000
C1_rand - C3_rand	1.120	72.813	58.125	0.263	1.000	1.000
C1_rand - C4_orig	-0.405	72.813	78.125	0.685	1.000	1.000
C1_rand - C4_rand	-0.238	72.813	75.938	0.812	1.000	1.000
C2_orig - C2_rand	-2.755	39.875	76.000	0.006	0.165	0.159
C2_orig - C3_orig	-0.977	39.875	52.688	0.329	1.000	1.000
C2_orig - C3_rand	-1.392	39.875	58.125	0.164	1.000	1.000
C2_orig - C4_orig	-2.917	39.875	78.125	0.004	0.099	0.099
C2_orig - C4_rand	-2.750	39.875	75.938	0.006	0.167	0.159
C2_rand - C3_orig	1.778	76.000	52.688	0.075	1.000	1.000
C2_rand - C3_rand	1.363	76.000	58.125	0.173	1.000	1.000
C2_rand - C4_orig	-0.162	76.000	78.125	0.871	1.000	1.000
C2_rand - C4_rand	0.005	76.000	75.938	0.996	1.000	1.000
C3_orig - C3_rand	-0.415	52.688	58.125	0.678	1.000	1.000
C3_orig - C4_orig	-1.940	52.688	78.125	0.052	1.000	1.000
C3_orig - C4_rand	-1.773	52.688	75.938	0.076	1.000	1.000
C3_rand - C4_orig	-1.525	58.125	78.125	0.127	1.000	1.000
C3_rand - C4_rand	-1.358	58.125	75.938	0.174	1.000	1.000
C4_orig - C4_rand	0.167	78.125	75.938	0.868	1.000	1.000

Table S11: Kruskal-Wallis rank sum test results of the tapping speed among the four conditions. In the table are listed the degree of freedom (*df*), chi-squared (χ^2), the p-values (*p*), and the eta-squared (ε^2) with the respective magnitude.

Signal	*df*	χ^2	*p*	ε^2	Magnitude
**Tapping speed**	3	2.69	0.44	0.005	small

Further Analysis - Tapping synchronization during a self-paced tapping paradigm

Table S12: Kolmogorov-Smirnov test results of the global tapping synchrony among the four conditions (real and random pairs). In the table are listed the Kolmogorov-Smirnov test statistics (*Z*), the *p*-values (*p*), and the effect size (*D*). In bold are shown the *p*-values that are significant after FDR correction.

Comparison	Z	*p*	*D*
$C1^{orig}$ - $C1^{rand}$	1	**< 0.001**	0.180
$C2^{orig}$ - $C2^{rand}$	1	**< 0.001**	0.180
$C3^{orig}$ - $C3^{rand}$	0.36	0.273	0.065
$C4^{orig}$ - $C4^{rand}$	0.53	**0.020**	0.090

Table S13: Kruskal-Wallis rank sum test results of the tapping coherence over the two frequency bands (unimodal auditory and bimodal) among the four original conditions. In the table are listed the degree of freedom (*df*), chi-squared (χ^2), the *p*-values (*p*), and the eta-squared (ε^2) with the respective magnitude. A single asterisk indicates $p < 0.05$ after the FDR correction.

Range	*df*	χ^2	*p*	ε^2	Magnitude
**Unimodal-auditory [2.17-7 Hz]**	3	43.21	**< 0.001** *	0.15	large
**Bimodal [0.5-2.17 Hz]**	3	41.87	**< 0.001** *	0.15	large

*** Significant *p*-values after the FDR correction**

Table S14: Post-hoc Dunn test results of the tapping coherence in the unimodal-auditory band among the four original conditions. In the table are listed the *z*-test statistic (*Z*), the *p*-values (*p*), and the Holm-adjusted *p*-values (*p*.adj). All the significant adjusted *p*-values are marked in bold typeface.

Comparison	Z	*p*	*p.adj*
C1 - C2	0.04	0.966	0.966
C1 - C3	2.63	0.008	**0.025**
C2 - C3	2.59	0.010	**0.019**
C1 - C4	5.70	0.001	**< 0.001**
C2 - C4	5.66	0.001	**< 0.001**
C3 - C4	2.98	0.003	**0.012**

Table S15: Post-hoc Dunn test results of the tapping coherence in the bimodal band among the four original conditions. In the table are listed the *z*-test statistic (*Z*), the *p*-values (*p*), and the Holm-adjusted *p*-values (*p*.adj). All the significant adjusted *p*-values are marked in bold typeface.

**Comparison**	**Z**	***p***	***p.adj***
C1 - C2	-0.08	0.940	0.940
C1 - C3	2.05	0.040	0.081
C2 - C3	2.12	0.034	0.101
C1 - C4	5.61	< 0.001	**< 0.001**
C2 - C4	5.68	< 0.001	**< 0.001**
C3 - C4	3.46	0.001	**0.002**

Section S1: Control analysis of tapping synchronization delivering evidence for variability of task execution.

The results from the control analysis yielded surprising findings (*see Figure S1*a). As anticipated, $C1^{rand}$ and $C2^{rand}$ produced significantly lower synchrony than their correctly paired counterparts $C1^{orig}$ and $C2^{orig}$ ($p < 0.001$, $D = 0.180$) and showed no difference to $C4^{orig}$, which is understood as random coherence. This could be viewed as a rejection of the null hypothesis by providing evidence that synchrony of correctly paired dyads is not random when sensory communication is possible. However, this could also be understood as proof of highly variable task execution across all dyads. During C1 and C2, correctly paired dyads produced unique tapping patterns that impeded random combinations from showing any comparable synchrony. In contrast, if all participants had synchronized to the same rhythm produced by a metronome, randomly formed dyads would have had similar coherence as the original. This highlights that a control analysis by randomization may be applied to investigate the similarity of task execution. And while control analysis alone could raise an uncertainty of whether its results relate to chance or task similarity, by implementing a further control condition, i.e. C4, chance findings can be ruled out. In condition 3 for instance, $C3^{rand}$ did not present with any significantly different tapping synchronization from $C3^{orig}$. By comparison to $C4^{orig}$, one can assume, that tapping synchrony for correct pairings was not random. Therefore, control analysis most likely indicates a similar task execution across all dyads. Our hypothesis for this emerging similarity in task execution during condition 3 will be further elaborated in Section S2.

As in C4, the control condition, expectedly presented with lower synchrony in $C4^{orig}$, it came as quite a surprise that $C4^{rand}$ produced significantly higher tapping synchronization ($p < 0.05$; $D = 0.090$). Certainly, with no possible communication between the individuals, $C4^{orig}$ could only represent random coherence. Thus, why randomly paired data produced higher synchrony than the original analysis and even showed no significant difference to both $C2^{orig}$ and $C3^{orig}$ is rather difficult to explain. One possible explanation could originate from the way control analysis was performed. For control analysis 60 virtual combinations were created and analyzed. Due to the comparably less engaging task of only tapping for themselves, individuals might have started to produce simpler and less variable rhythms. During the original analysis, this coincidentally did not create higher synchrony but when control analysis created these 60 virtual dyads, some of these tapping patterns accidentally must have aligned very strongly, while others did very little or not at all. This could explain why the mean coherence index is rather low, but the overall variability is very large.

To sum up, the control analysis of the tapping synchronization data can be used to evaluate similarities of task execution across all participants. With this, we could demonstrate that during conditions 1 and 2 the performed tapping rhythms differed significantly between the original dyads, while during condition 3 and also during condition 4 more similar tapping rhythms were chosen.

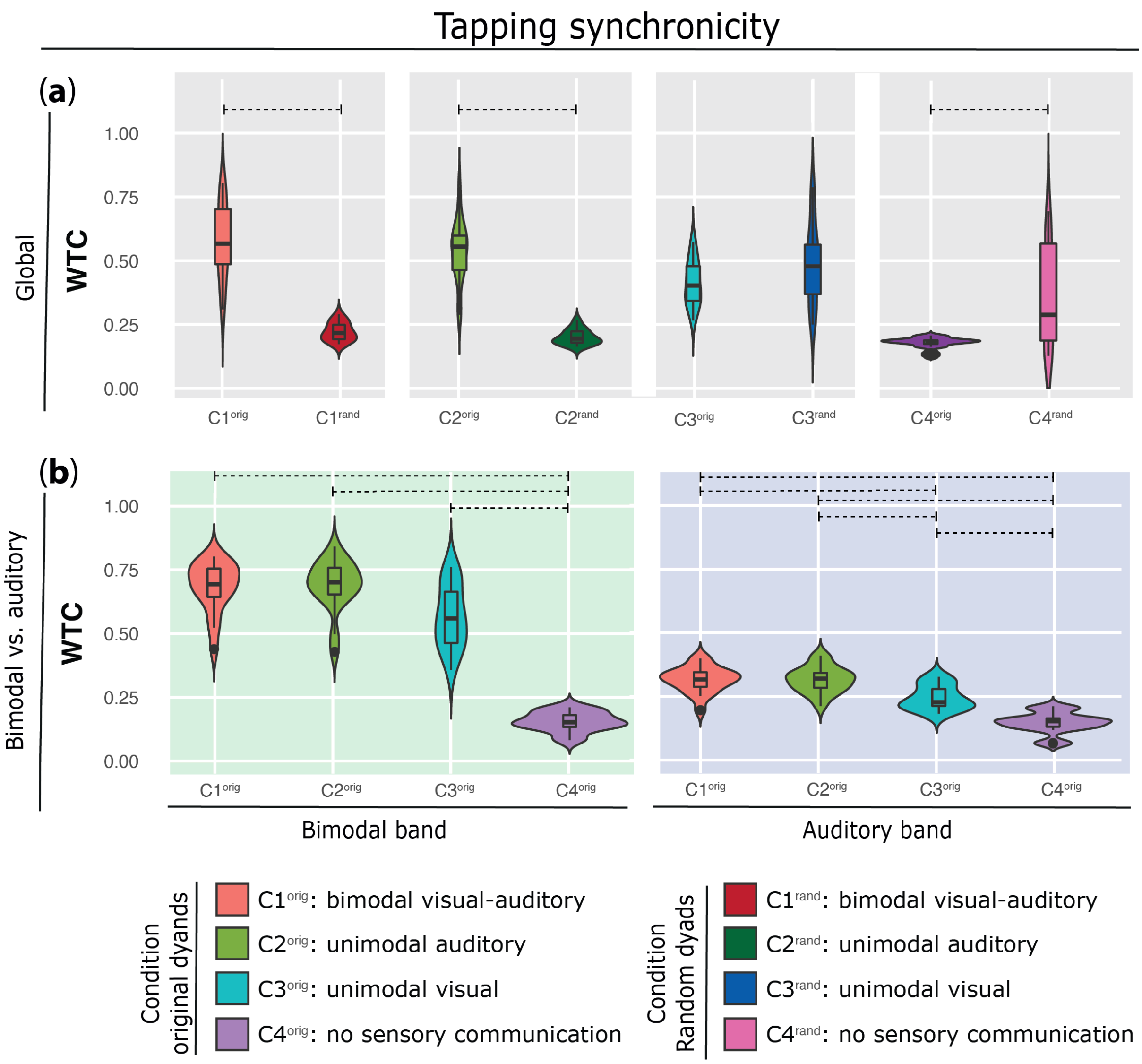

Figure S1 Tapping synchrony according to conditions (a) Control analysis of randomized and original dyads per condition. (b) Tapping synchrony of bimodally (0.5–2.17 Hz) and unimodally auditorily (2.17–7 Hz) accurately processable tapping frequencies according to original conditions. Comparisons marked with dotted bars express significant differences ($p < 0.05$).

Section S2: Self-paced tapping paradigm potentially adds new interesting behavioral component to motor entrainment research

How accurate motor entrainment can be performed depends in part on the sensory modality by which the pacing rhythm is transmitted. Studies investigating the effectiveness of both auditory and visual stimuli demonstrated that auditory rhythmical perception showed consistent superiority[1-4]. There was even a tendency to focus on auditory stimuli when confronted with both[1]. These findings are at least partially attributed to the auditory rhythm perception's superior temporal resolution[1,2] and its more efficient phase correction mechanism[2]. When comparing the performance of bimodal rhythmical stimuli (i.e. auditory and visual combined), against unimodally transmitted periodicities (i.e. auditory or visual separate), it was shown that both auditory and visual modes combined produced an even more accurate motor entrainment[5]. These findings suggest that all sensory modalities play an important part in the accurate timing and synchronization of human behavior.

Interestingly, however, as displayed in *Figure 1 (see article)*, our results show deviations from these previous results. During unimodal-auditory communication they did not yield higher synchrony than unimodal-visual (p = 0.065, ε^2 = 0.597), while bimodal communication was significantly more accurate than unimodal-visual (p = 0.031). This could imply that our brain processes bimodally perceived rhythms more accurately than visually perceived while auditory processing alone shows no clear superiority over unimodal-visual. However, given the substantial evidence of the superiority of auditory over visual rhythm processing[1-4] and these borderline significant p-values (both of them being significant before Holm's correction), we rather suggest other reasons for these findings. Firstly, it is possible that these particular results would become significant if more subjects were included. Secondly, we chose a procedure that allowed followers to fully see the senders' hands. Contrary to other experiments[1,2,4,5], where the visual timing cue was a sudden flash of light on a computer screen, in our paradigm followers could observe the whole movement of the finger and, thus, were able to anticipate the taps. This could certainly facilitate tapping synchronization during unimodal-visual communication. However, we believe there to be yet another potential reason for our results. Contrary to these studies with external steady pacing[1-5], we applied a self-paced tapping paradigm. This added a new behavioral dynamic to the experiment. Each sender was allowed to choose a rhythm to their liking. As inhibition of certain communication channels seems to create disadvantages for accurately tapping in synchrony, this implies that, generally, whenever a more proficient sensory modality was blocked the difficulty to maintain tapping synchronization accuracy increased. With the given aim to maximize synchronization, participants thus had both the opportunity as well the motivation to actively counteract increasing difficulties. We believe this led to mutual behavioral adjustments facilitating synchronization: participants helped each other out by choosing simpler rhythm patterns, slowing down, and mutually adjusting their tapping to each other. This process, which we consider *cooperative counterbalancing,* could have contributed to higher synchrony in unimodal-visual communication due to the production of easier tapping rhythms even though they were perceived by a less accurate sensory modality.

This notion finds support when looking at the results of the control analysis (*see Figure S1*a). Since no significant difference between unimodal-visual $C3^{orig}$ and $C3^{rand}$ could be demonstrated, this indicates a similar task execution across all dyads. We believe the emerging similarity in task execution during condition 3 resulted from participants choosing to perform simpler and more isochronous tapping rhythms to achieve maximal synchronization. This resulted in a lower tapping rhythm variability across all dyads, specifically leading to the emergence of more homogenous tapping patterns. These findings thus strongly point towards cooperative counterbalancing. Furthermore, Lorås et al. [6] demonstrated that side-by-side seated individuals spontaneously aligned their tapping to each other, even if no aim to synchronize was given. The inherent tendency of interacting individuals to mutual entrainment, which was displayed in their study, could serve as a foundation for cooperative counterbalancing. To sum up, we provide evidence indicating that during a self-paced interpersonal finger tapping synchronization task sensory communication channel blocking provokes participants to adjust their tapping rhythm to achieve higher tapping synchrony.

Contrary to Elliott et al.[5], we did not provide evidence for the higher accuracy of bimodal compared to auditory perception, as no significant difference was found in the global tapping coherence analysis in both frequency bands. Yet as mentioned above, these results may only be in part comparable to our findings. By using a self-pacing approach, we not only studied the dependency of motor entrainment on the sensory modality but also how behavior in real-life human interaction may affect this process. This could lead to questioning the relevance of certain sensory communication channels. Lorås et al.[6] presented the idea, that in self-paced

interpersonal motor entrainment tasks the interacting individuals display a tendency to produce fast, visually not processable frequencies. Therefore, during bimodal communication, individuals may rely predominantly on auditory communication channels. This hypothesis finds further support by research on spontaneous tapping speeds[7] which demonstrated that spontaneous tapping already approached frequencies very close to the upper limit of visual processing (≈2.17Hz) while still posing no challenge to auditory processing (≈ 9Hz)[8]. Additionally, when tapping in synchrony with someone else, tapping frequencies were shown to spontaneously increase, potentially due to the arousal experienced from social interaction[6] Our results, however, do not support Lorås et al.'s [6] idea, since average tapping speeds across all conditions did not express any significant difference and failed to exceed visual rhythmic processing speed. Additionally, according to their logic, the only hindrance to a more accurate bimodally mediated tapping synchronization is the human tendency to spontaneously tap "too fast". Thus, if tapping speeds of lower, visually processable frequencies were produced, visual processing ought to be adding additional accuracy to the synchronization. However, as displayed in *Figure S1*b, we could not show any significant difference in coherence from bimodal to unimodal-auditory communication even in the visually processable frequency band (0.5-2.17Hz). We, therefore, conclude that during self-paced interpersonal motor entrainment bimodal communication shows no superiority over unimodal-auditory. Moreover, we have reason to believe that this is due to the superiority of auditory rhythm processing which leads to neglection of visual input when confronted with both.

During the control condition ($C4^{orig}$), where no sensory modality was left to communicate, thus disabling any rhythm perception and cooperative counterbalancing, synchrony significantly decreased compared to all other conditions in global tapping synchrony ($p < 0.001$; except for C3-C4 $p = 0.004$) as well as both unimodal-auditory ($p < 0.001$; except for C3-C4 $p = 0.012$) and bimodal frequency bands ($p < 0.001$; expect C3-C4 $p = 0.002$). This demonstrates that without any sensory communication, interindividual tapping synchronization is not possible.

References for supplementary information

1 Repp, B. H. & Penel, A. Rhythmic movement is attracted more strongly to auditory than to visual rhythms. *Psychol Res* **68**, 252-270 (2004). https://doi.org:10.1007/s00426-003-0143-8

2 Repp, B. H. & Penel, A. Auditory dominance in temporal processing: new evidence from synchronization with simultaneous visual and auditory sequences. *J Exp Psychol Hum Percept Perform* **28**, 1085-1099 (2002).

3 Hove, M. J., Iversen, J. R., Zhang, A. & Repp, B. H. Synchronization with competing visual and auditory rhythms: bouncing ball meets metronome. *Psychol Res* **77**, 388-398 (2013). https://doi.org:10.1007/s00426-012-0441-0

4 Lorås, H., Sigmundsson, H., Talcott, J. B., Ohberg, F. & Stensdotter, A. K. Timing continuous or discontinuous movements across effectors specified by different pacing modalities and intervals. *Exp Brain Res* **220**, 335-347 (2012). https://doi.org:10.1007/s00221-012-3142-4

5 Elliott, M. T., Wing, A. M. & Welchman, A. E. Multisensory cues improve sensorimotor synchronisation. *Eur J Neurosci* **31**, 1828-1835 (2010). https://doi.org:10.1111/j.1460-9568.2010.07205.x

6 Lorås, H., Aune, T. K., Ingvaldsen, R. & Pedersen, A. V. Interpersonal and intrapersonal entrainment of self-paced tapping rate. *PLoS One* **14**, e0220505 (2019). https://doi.org:10.1371/journal.pone.0220505

7 McAuley, J. in *Music perception* (eds M. R. Jones, R. R. Fay, & A. N. Popper) Ch. 165–199, (Springer-Verlag New York, 2010).

8 Repp, B. H. Rate limits in sensorimotor synchronization with auditory and visual sequences: the synchronization threshold and the benefits and costs of interval subdivision. *J Mot Behav* **35**, 355-370 (2003). https://doi.org:10.1080/00222890309603156
